# Considerations in Surgical Management of Pediatric Obstructive Sleep Apnea: Tonsillectomy and Beyond

**DOI:** 10.3390/children8110944

**Published:** 2021-10-20

**Authors:** T. C. Uwiera

**Affiliations:** 1 Department of Surgery, Faculty of Medicine and Dentistry, University of Alberta, Edmonton, AB T6G 2B7, Canada; tuwiera@ualberta.ca; 2 Pediatric Otolaryngology, Stollery Children’s Hospital, Edmonton, AB T6G 2B7, Canada

**Keywords:** tonsillectomy, intracapsular tonsillectomy, pediatric, obstructive sleep apnea, surgery, supraglottoplasty, epiglottopexy

## Abstract

Obstructive sleep apnea (OSA) is an increasingly recognized disorder with a reported incidence of 5.7% in children. Tonsillectomy (with or without adenoidectomy) in pediatric OSA in otherwise healthy non-obese children has a success rate of approximately 75%. However, the cure rate reported for all children undergoing tonsillectomy varies from 51% to 83%. This article reviews the history of tonsillectomy, its indications, techniques, various methods, risks, and successes. The article also explores other surgical options in children with residual OSA post-tonsillectomy.

## 1. Introduction

Obstructive sleep apnea (OSA) in children is an increasingly recognized condition. Management options include observation, weight loss, medical management, management of allergies or underlying conditions, non-invasive home ventilation, surgery, or some combination of these. Surgical management typically includes tonsillectomy, with or without adenoidectomy to beneficial effect. In children with persistent or residual OSA, however, other surgical procedures beyond tonsillectomy may be required. This article explores the history, indications, techniques, methods, complications of tonsillectomy, and beyond.

## 2. Tonsillectomy in History

Tonsillectomy, the surgical removal of a portion or the entire tonsil along with the surrounding capsule has been completed for centuries. The first reported tonsil surgery is documented in 700 BC [1,2] in a Hindu Sanskrit document Atharva-Verda. It appears again in 50 AD described by Celsus [3,4]; however, it was not practiced widely given the risk of significant hemorrhage [2,4]. The development of the guillotine tonsillectomy in 1827 permitted rapid extraction of the tonsil [4]; however, prior to development of anaesthesia in the 1840s [5], there was not a wide adaptation of this procedure. With the emergence of anaesthesia, surgeons were able to improve the procedure by slowly dissecting tissue in a controlled fashion [5] prompting the development of the dissection tonsillectomy in 1917 [4]. From that time, tonsillectomy as a recognized procedure gained prominence, with tonsillectomy now being one of the most common surgical procedures [6,7,8] in children.

Tonsillectomy has been controversial through the ages sparking the New York “tonsil riots” in 1906 [9] when tonsillectomies were completed on school-aged children for recurrent upper respiratory infections. Articles in newspapers and medical literature have dubbed tonsillectomy “A Wicked Operation” [10] questioning “Are all ‘T&As’ Really Necessary” [3]. This “tonsil problem” remains hotly contested in contemporary medical practice both for the indications to perform the surgery, and the choice of the “best” technique of surgery.

## 3. Changing Indications for Tonsillectomy

The indications for tonsillectomy at the turn of the century varied from anorexia, rheumatism, nephritis, “mental retardation”, and enuresis [1,3]. In the pre-antibiotic era, tonsillectomy was used to treat deep neck abscesses, septic emboli, diphtheria, poststreptococcal glomerulonephritis, and rheumatic fever [4]. This approach was enthusiastically adapted by the 1930s for “catarrhal” children resulting in tonsillectomy accounting for one third of all surgical procedures in the United States [3] and removal of 50–75% of all British children’s tonsils [3,4]. With the advent of antibiotics, however, by the 1960s tonsillectomy was predominantly reserved to treat recurrent acute tonsillitis [1].

Indications for tonsillectomy were further refined in subsequent decades, as Paradise et al. published stringent eligibility criteria in 1984 limiting tonsillectomy for severe and recurrent infections [11]. These criteria are still in use today [12]. Tonsillectomy for OSA appeared in the mid-1970s when OSA was recognized as a condition that not only affected adults, but children as well [4]. The current and most frequent indications for tonsillectomy (with or without adenoidectomy) include recurrent tonsil infections that meet the Paradise criteria [11] and sleep disordered breathing or obstructive sleep apnea symptoms [12,13,14].

Over the last century, the impetus to remove tonsils to prevent infections or their associated complications has been surpassed with surgery for obstructive breathing symptoms. In a study by Rosenfeld and Green [4] at a tertiary center in the USA, the indications for tonsillectomy changed dramatically between 1978 and 1986. In 1978, 100% of tonsillectomies were for recurrent infections. By 1986, this decreased to 81% with obstructive symptoms accounting for the remaining 19% [4]. Erickson et al. further characterized this shift [15] noting 12% of patients underwent tonsillectomy for obstructive symptoms in 1970, increasing to 77% in 2005. Despite this observation, indications for tonsillectomy remain fraught with challenges as there can be marked practice variation at local, regional and international levels [1,16,17].

## 4. Obstructive Sleep Apnea Diagnostic Dilemmas

Obstructive sleep apnea in children has a reported incidence of up to 5.7% in childhood [16,18,19] and in obese children, the prevalence rises to 60% [20]. This is a spectrum of disease ranging from primary snoring, upper airway resistance syndrome, to OSA. Primary snoring is defined as snoring without medical comorbidity [21]. Upper airway resistance syndrome was coined in 1982 to encompass increasingly negative intrathoracic pressures on inspiration resulting in arousal, sleep fragmentation without perceived apneas, hypopneas, or oxygen desaturations [19]. Finally, OSA is defined by the American Thoracic Society as “a disorder of breathing during sleep characterized by prolonged partial upper airway obstruction and/or intermittent complete obstruction that disrupts normal ventilation during sleep and normal sleep patterns” [22]. While there is increasing awareness among primary care physicians and otolaryngologists to both recognize and treat pediatric OSA, controversy remains on how to accurately diagnose OSA in this population.

Despite numerous published guidelines and recommendations, significant variation remains in the diagnosis of OSA in children. Polysomnography (PSG) is considered the gold standard for diagnosis of OSA. An apnea-hypopnea index of >1 in the PSG is considered to be positive for OSA in children [22]. Other respiratory and non-respiratory markers of OSA in children are well reported [22], but are beyond the scope of this article. The challenge comes in how infrequently PSG is used to diagnose OSA in clinical practice. Radhakrishnan et al. [23] explored how often PSG was completed in children <10 years of age where cost of the PSG was not a consideration (universal access to health care system). Of the 27,837 children undergoing adenotonsillectomy for OSA over a ten-year period, only 12.8% had a PSG within 18 months prior to, and 5.7% had a PSG within the 12 months following surgery.

Other measures for determining OSA in lieu of PSG have been examined. These surrogate measures of OSA include tonsil size [13,24,25] clinical assessment, questionnaires [24,26], sleep videos [23], and overnight oximetry [14,24,27], with notable variables and often contested capacity to accurately identify children with OSA. As such, there is currently little consensus on the best way to define pediatric OSA that is widely applicable, accessible, successful, and cost effective when considering surgical management to treat the disease.

## 5. Surgical Management of OSA

Adenotonsillectomy, the removal of the adenoids and tonsils, is the primary treatment for pediatric OSA [13,28,29,30]. It is difficult to only assess children who undergo tonsillectomy, without adenoidectomy, as the literature often does not distinguish between adenotonsillectomy and tonsillectomy alone [6,12,14,15,16]. There is little evidence to support adenoidectomy for treatment of recurrent tonsil infections [14]; however, for obstructive symptoms, tonsillectomy or adenotonsillectomy are often discussed interchangeably [14,15,16]. As discussed above, the surgical indications for tonsillectomy have shifted from infections to obstructive symptoms over the last several decades. Tonsillectomy remains one of the most common surgeries for children.

### 5.1. Anatomy of the Tonsil

A thorough understanding of the anatomy and blood supply of the tonsils is essential in safe surgical dissection and control of hemorrhage. Waldeyer’s ring of lymphoid tissue in the nasopharynx and oropharynx is comprised of the palatine tonsils, the adenoids, the tubal tonsils, and lingual tonsil. The palatine tonsils reside between the palatoglossus (anteriorly) and palatopharyngeus (posterior) muscles laterally. There is a fibrous capsule that demarcates the palatine tonsil from the surrounding musculature with a potential peritonsillar space between the two. The tonsil capsule may be disrupted by infection of the tonsil itself or by peritonsillar abscesses. The tonsillar blood supply is rich with vessels from the external carotid artery, namely, the lingual, facial, ascending pharyngeal, and internal maxillary arteries with several branches from these. The robust arterial blood supply to the tonsils accounts for the significant perioperative risk of bleeding.

### 5.2. Surgical Techniques and Methods of Tonsillectomy

Surgical technique of tonsillectomy refers to whether the entire palatine tonsil is removed, total tonsil and capsule removed in total tonsillectomy (TT), or whether the capsule or portion of the tonsil is left in situ known as intracapsular tonsillectomy (IT). The method refers to what instruments are used to remove the tonsil in whole or part. There are many different methods to perform tonsillectomy. These are divided into “cold” or “hot” methods. “Cold” methods, where no heat is used, include cold steel dissection, guillotine, microdebrider, harmonic scalpel, and plasma blade. “Hot” methods include electrocautery (monopolar or bipolar), coblation (radiofrequency-controlled ablation), or laser [2,8,31,32,33,34,35]. To date, no single technique has been widely accepted as the most superior method for performing the surgery [2,31,36,37]. Indication for surgery also does not determine the method of tonsillectomy [8]. The technique chosen by surgeons varies with a surgeon’s experience and comfort level with any specific technique [6]. Additionally, other factors such as cost, historic practice, or equipment availability may influence a surgeon’s choice.

#### 5.2.1. Total Tonsillectomy vs. Intracapsular Tonsillectomy

In total tonsillectomy (TT), also known as extra-capsular tonsillectomy, the tonsil and its surrounding capsule are removed completely [32,33,34]. This leaves bare pharyngeal musculature in the tonsillar fossa to heal by secondary intention [35,38]. Intracapsular tonsillectomy (IT), also known as partial tonsillectomy, subtotal tonsillectomy, or tonsillotomy, removes the majority of the tonsillar tissue but leaves the capsule surrounding the palatine tonsil intact [33,35,38]. In this technique, the pharyngeal muscle is not exposed and in theory there is less post-operative pain and risk of hemorrhage.

#### 5.2.2. Post-Operative Complications in Total Tonsillectomy vs. Intracapsular Tonsillectomy

Post-tonsillectomy pain and hemorrhage in IT versus TT has been examined repeatedly in an effort to determine the “best” technique. There is general agreement that IT has less post-operative hemorrhage than TT [32,33,39]. Unfortunately, there is considerable disparity between the studies available, making direct comparisons challenging. Many studies compare various IT to TT techniques, but the method of surgery, pain scores, and the amount of blood needed to qualify as post-operative hemorrhage also vary between studies. Daskalakis et al. completed a systematic review and meta-analysis in 2021 examining coblation IT versus coblation TT. They found only six studies available to date for comparison, and even among those studies the variation posed a challenge to draw definitive conclusions. In their review, they found post-operative bleeding was higher in the TT vs. IT by coblation groups. There was a significant difference in late post-operative pain between the groups, with IT being less painful at the late assessment. There was no significant difference in early post-operative pain between the groups [33].

#### 5.2.3. Tonsillar Regrowth Resulting in Recurrent OSA and Repeat Tonsillectomy

Regrowth of tonsillar tissue is a potentially unwelcome consequence of IT should the tonsil once again cause symptoms. The literature reports a six fold higher risk of residual tonsillar tissue after IT versus TT [39]. Risk of tonsillar regrowth with recurrence of OSA following IT ranges from 0% to 16.6% depending on the method of IT [39]. The overall rate of regrowth has proven difficult to quantify as many studies do not follow children long term [13,39]. Keltie et al. noted that the rate of revision tonsillectomy was double after coblation compared to tonsillectomy by cold dissection in a five year period (1.4% and 0.6%, respectively). They proposed this could be due to the learning curve of the newer coblation technique and more use of the IT technique [32]. In one study within a 15-year period, the revision rate for tonsillectomy after IT was 1.39%. Sagheer et al. noted that age <5 years at the time of surgery, a history of gastroesophageal reflux, or a history of tonsillitis were associated with the need for revision tonsillectomy [39]. Tonsil tissue is most immunologically active between the ages of 3 and 10 years [12]. It is postulated that the younger the child at the time of tonsillectomy, the greater the time of ongoing immunologic activity with the greatest risk of tonsillar regrowth. Longitudinal data is still needed to determine if IT is widely applicable for surgical treatment of pediatric OSA.

#### 5.2.4. “Cold” Tonsillectomy Methods

##### Cold Steel Tonsillectomy

The cold steel tonsillectomy is completed with reusable, resterilizable metal surgical instruments. The palatine tonsil is dissected free (total tonsillectomy completed) of the peritonsillar space and bleeding is controlled by ligation or electrocautery [8,35]. Reusable metal instruments are commonly used in surgery; however, in the United Kingdom (UK) in the 1990s, this practice was brought into question with concerns of spreading Creutzfeldt–Jakob disease between patients. Creutzfeldt–Jakob disease is a neurodegenerative disorder due to prion infection [40]. Prions are resistant to standard sterilization methods, hence raising concern of infecting patients with contaminated equipment. In response to this concern in 2001, the Department of Health in the UK recommended single-use, disposable equipment for tonsillectomies and other surgical procedures [41,42]. The concern of prion transmission in tonsillectomy was later dismissed after several studies examining tonsil specimens failed to discover prions in the tonsil tissue, nor risk of Creutzfeldt–Jakob disease in previous tonsillectomy patients [40,43].

Cold steel tonsillectomy is still the one most commonly used methods around the world, especially in the developing world [2]. It is thought to be more time consuming than other methods; however, this has not always been substantiated in the literature [44,45]. It is associated with more intra-operative blood loss than other methods but is thought to cause less tissue damage and be less painful post-operatively [8,44]. It is the least costly method as the instruments can be sterilized and reused, which may account for its use in developing countries [2]. This is the standard or traditional tonsillectomy method to which other methods are often compared.

##### Guillotine

Guillotine tonsillectomy, or removal by tonsillotome, was described in 1827 [2,4] to remove the portion of the tonsils (IT only) that could be pulled away from the tonsillar fossae and excised. It was the predominant method for over 80 years; however, it often failed to resolve symptoms of infections as a portion of the tonsil and the capsule remained in situ [2]. It is no longer in common use in favor of other methods.

##### Microdebrider

Microdebriders, powered soft tissue shavers, were introduced in 2002 [2] after adapting the tool’s use from arthroscopic orthopedic surgery. This method uses a single use disposable handpiece and is typically used for IT techniques removing 90–95% of the tonsillar tissue. There is a physiologic “bandage” left overlying the musculature [2], which is thought to decrease post-operative hemorrhage and pain [46]. Hemostasis is generally achieved after IT with electrocautery. The resultant costs of this surgical method are higher than other methods as both the shaver handpiece and an electrocautery device are required [46].

##### Plasma Blade

This is a newer single use device that uses radiofrequency to cut and coagulate tissue. It creates a highly ionized plasma field around an electrode using the surrounding tissue electrolytes. It has lower average temperatures (40–100 °C) than traditional monopolar cautery (200–600 °C). This is thought to cause less thermal damage resulting in less tissue damage, less post-operative pain, and faster wound healing [34,44]. There have been reports, however, of difficulty controlling bleeding intra-operatively. Some studies have not found any difference in rates of wound healing [44]. In a retrospective study in 2021 by Chen and Chen, they found no significant difference in post-tonsillectomy hemorrhage rates in comparison to monopolar cautery [34], and a randomized controlled trial (RCT) in 2017 found no difference in post-operative hemorrhage compared to cold steel [44]. There was no significant difference in surgical time with the plasma blade versus cold steel in the RCT [44]. The cost for this single use item is high and exceeds that of coblation [44].

#### 5.2.5. “Hot” Tonsillectomy Methods

##### Electrocautery

Electrocautery delivers radiofrequency energy via an instrument providing kinetic energy that heats the intracellular and extracellular fluids and ruptures localized tissue [2,8]. This uses an electrically powered reusable handpiece with a variety of disposable tips (blade or needle variations). This generates heat that may reach 300–440C. It is thought to cause more thermal damage to the surrounding tissue that may contribute to post-operative pain [2]. The disposable costs for electrocautery are low compared to coblation (USD 0.28 and USD 320 respectively) [47]. In the United States, monopolar electrocautery is the most popular method of tonsillectomy [6].

##### Coblation

Coblation, or radiofrequency controlled ablation, uses an electrically powered single use handpiece with saline irrigation to create an ionized plasma field. This plasma field has energetic charge-carrying ions with sufficient energy to break organic molecular bonds resulting in the breakdown or “ablation“ of the tissue [6,35,41]. The heat generated in this method is less than in electrocautery at 40–70C [8]. There was initially significant excitement with this method as initial studies showed decreased post-operative pain and hemorrhage. A Cochrane review in 2017, however, found that the evidence for a difference in post-operative pain compared to other TT methods is of low or very low quality. They also reported the evidence for a difference in post-tonsillectomy hemorrhage is also of low quality [35].

Coblation remains a common method and can be used for TT or IT. Keltie et al. documented a change in clinical practice in England from 2008 to 2019. Initially coblation was only used in pediatric tonsillectomy in 7% for the first year of study. By 2019, however, the proportion of tonsillectomies by coblation had increased to 27% [32]. Research is ongoing to determine if the increased cost of the single use coblation handpiece is justified by cost savings in length of hospital stay, return to hospital with pain or bleeding, and risk of revision tonsillectomy [47]. 

##### Laser

Laser was introduced as a concept for a bloodless tonsillectomy in 1994. Both CO_2_ and KTP lasers have been used in either TT or IT. While there was less intra-operative bleeding, this method fell out of favor due to increases in both post-operative pain and secondary hemorrhage [2].

## 6. Choice of Surgical Method for Tonsil Surgery

Choosing the method of tonsillectomy is done in a variety of ways. Often this is left to the surgeon to determine based on his or her experience, proficiency, and results with a specific method [48,49]. Occasionally, patients select the method, either directly by choosing the device of preference [34], or indirectly when choosing a surgeon with a preferred method. Cost may be a consideration for patients, surgeons, and institutions. McCoy et al. looked beyond the disposable costs of the equipment, examining the entire hospital stay including: equipment costs, surgical time, recovery period, analgesic use, and return to hospital for peri-operative complications. They noted that at their institution, despite a huge cost variation in the disposable electrocautery versus coblation devices (USD 0.28 vs. USD 320), the overall cost of the entire procedure and recovery may be comparable [47]. Meiklejohn and Chavarri [31] argue that when surgical outcomes (success or complications) do not differ between different methods of tonsillectomy, surgeons should consider the method that generates the least waste and is the most cost effective. They found at their institution, the costs of each method varied considerably, with cold steel, monopolar electrocautery, and coblation being USD 17.51, USD 27.76 and USD 203.46 per case, respectively. The amount of disposable waste produced, as all hospital waste has a disposal cost, was also least with cold steel and the most with coblation. Changing from coblation to cold steel for tonsillectomy, given the number of procedures completed annually worldwide, could have a significant impact on both the economic and environmental costs.

## 7. Risks of Tonsillectomy

While tonsillectomy is a common procedure, it has potential risks that are well recognized by clinicians. There are risks related to anaesthesia, to the procedure itself, and events during the post-operative period. Anaesthetic concerns include adverse medication reactions, difficult intubation, laryngospasm, laryngeal edema, aspiration, respiratory compromise, endotracheal tube ignition, and cardiac arrest. Operative complications include injuries to the teeth, lips, larynx, pharyngeal wall, or soft palate. Injuries to adjacent tonsillar structures including the carotid artery, fracture of the mandibular condyle, tongue swelling, uvular edema, altered taste, or orbital injury are possible. The most common post-operative complications include nausea, vomiting, pain, and bleeding. Others include dehydration, referred otalgia, post-obstructive pulmonary edema, velopharyngeal insufficiency, and nasopharyngeal stenosis [2,8,12,35,50].

Given the risk of perioperative complications, it is recommended that some children be monitored post-operatively. These include children <3 years of age, those with complex histories such as Trisomy 21, neuromuscular disorders, craniofacial anomalies, obesity (BMI > 95th percentile for age), those with severe OSA (defined as an AHI ≥ 10 or oxygen < 80% or both), or those children whose behavioural factors may predict poor oral intake or difficult pain management are recommended to be observed post-operatively [12]. While there is consensus on who should be monitored perioperatively, there is little agreement on what type of monitoring is needed. Protocols include oximetry monitoring in the post anaesthetic recovery unit (PACU), on a short stay or day unit, in a general care bed, intensive care step down unit, or the PICU [12].

### 7.1. Respiratory Complications

Respiratory complications can occur intra-operatively, in the PACU, or post-operatively. Major respiratory complications include bronchospasm, laryngospasm, post-obstructive pulmonary edema, airway obstruction, aspiration pneumonitis, pneumonia, or cardiopulmonary arrest [8,51]. These events typically result in reintubation, CPAP/BIPAP therapy, placement of a nasopharyngeal or oropharyngeal airway, bag mask ventilation, an unplanned admission of the patient to the hospital, elevation of care to the intensive care unit (ICU), pulmonary edema, or, rarely, death [52]. These reported major respiratory events vary from 5.8% overall [52] with up to 8% of events occurring intra-operatively and 5.7% in the post-operative period [8]. Minor respiratory events include hypoxemia that may require supplemental oxygen or may resolve spontaneously [8]. Trying to evaluate the risk of hypoxic events throughout the literature is complicated with the varied definitions of “hypoxia”, with some studies including any events under 95% oxygen saturation and others including only events less than 90%. There are certain patient specific risk factors for hypoxemia within the first 24 h post-operatively that have been identified. These include patients with Trisomy 21, obesity, age, black race, coexistent cardiac disease, clinical diagnosis of OSA, coexistent neurologic disease, or a prior diagnosis of pulmonary disease [50,51,53]. Given the lack of standardization in the literature defining what exactly characterizes a hypoxic event, the reported incidence varies from 5% to 30%, with a meta-analysis reporting a 9.4% overall incidence [50].

### 7.2. Hemorrhage Post-Tonsillectomy

Post-tonsillectomy hemorrhage is well studied and is determined as either primary bleeding (within 24 h of surgery) or secondary bleeding (>24 h up to 14 days post-operatively). Primary bleeding occurs from 0.2% to 2.2% following surgery, while secondary bleeding happens from 0.1% to 3% post-operatively [12]. How much bleeding (volume) constitutes a post-tonsillectomy hemorrhage, whether the child is observed in a clinical setting or at home, and how to accurately collect these data points is challenging. The heterogeneity of the data available makes a standard post-tonsillectomy protocol for hemorrhage difficult [12]. Xu et al. noted the method of tonsillectomy and surgical experience <5 years increased the risk of primary post-tonsillectomy hemorrhage [7]. A surgeon’s experience with a new method of tonsillectomy has also been noted to change risk of post-operative hemorrhage. Following recommendations in the UK in 2001 in response to concerns of Creutzfeldt–Jakob disease, there was wide use of single use coblation and electrocautery methods. In the initial year of the review, there was a three-fold increase in post-tonsillectomy hemorrhage with these new surgical methods compared to the familiar cold steel tonsillectomy [42]. The risk of bleeding was later found to be similar between cold steel, electrocautery and coblation as surgeons gained experience and proficiency with the newer methods [41]. In children presenting with post-operative hemorrhage, Xu et al. noted 6.52% presented with multiple bleeding episodes requiring surgery [7].

A recent publication suggests reported post-tonsillectomy hemorrhage rates may be underestimating the actual risk. Dhaduk et al. evaluated post-tonsillectomy hemorrhage at a national level over a 12-year period in the United States using the Kids Inpatient Database (KID), the largest publicly available all-payer pediatric inpatient care database in the United States [54]. They noted an overall post-operative bleeding rate of 11.9% of the nearly 46,000 cases completed across the nation in that period. Patients 6–17 years presented with post-tonsillectomy bleeding more often than those <6 years of age [55]. This has been previously noted with children >12 years having a 1.5–3-fold increase in primary bleeding after tonsillectomy [56]. Dhaduk et al. also noted that children with pre-existing anemia had an increased rate of post tonsillectomy bleeding [55]. There was also an increased risk of post-operative hemorrhage in white patients, those with a history of coagulopathies, or fluid and electrolyte disturbances. Dhaduk et al. postulated that the rate of post-tonsillectomy bleeding may be underestimated in the literature as often studies only examine a single institution with a few participating surgeons. Single site studies may fail to capture children presenting with post-operative hemorrhage to a different hospital. Single site studies may also minimize the variation between surgeons and between surgical methods [55]. Further multicenter, national studies are needed to fully understand the risk of post-tonsillectomy hemorrhage.

### 7.3. Mortality Post-Tonsillectomy

Death after tonsillectomy can occur from various complications. Severe hemorrhage is associated with one third of all deaths [12], while the remainder are related to aspiration, cardiopulmonary failure, electrolyte imbalance, or anaesthetic complications [12]. Mortality rates following tonsillectomy, although low, are not uniform and can vary within regions and between countries. Within one region in Canada from 2002 to 2013 the reported mortality was 0.0018% [12], while Sweden reported 0.0024% during 2004–2011 [57], England reported 0.0037% in 2008–2019 [32], with the United States reporting 0.0055% in 2010 [12]. Overall, the risk of death post-tonsillectomy remains very low; however, as this is generally an elective surgical procedure, the overall risk needs to be assessed in the context of risk versus expected benefit and outcomes post-operatively.

### 7.4. Persistence of OSA Post-Tonsillectomy

Tonsillectomy is the first line treatment for children with OSA. Resolution of OSA post-tonsillectomy (with or without adenoidectomy) in otherwise healthy non-obese children is approximately 75% [13]; however, the cure rate reported for all children undergoing tonsillectomy varies from 51% to 83% [28]. The Childhood Adenotonsillectomy Trial (CHAT) randomized children with OSA in either surgical management or surveillance with medical management. Children with surgery had improved outcomes with 79% resolution of their symptoms, compared to 46% in the medical management group [18]. This study supports the consensus among experts that adenotonsillectomy should be the first line treatment for OSA in healthy children aged 2–19 years. Most experts also agree (89% consensus) adenotonsillectomy is warranted in children <2 years, for both obese and non-obese children [13,58]. In children with Trisomy 21 or craniofacial disorders, adenotonsillectomy likewise remains the first line of therapy in 85% and 74% of the incidence respectively [13]. Finally, most studies also report significant improvements in respiratory parameters [28]. Many studies have shown persistent improvements in quality of life scores (from questionnaires: OSA-18, or Pediatric Sleep Questionnaire) at least 2 years following adenotonsillectomy [13,14,28].

Trying to accurately define what is persistent OSA post tonsillectomy (with or without adenoidectomy) is a challenge. Again, the definition of persistence of OSA varies across the literature often with few objective pre-operative measures to compare to post-surgical outcomes [28]. Additionally, the diagnostic dilemmas mentioned above contribute to the difficulty of objectively measuring changes after surgery. Despite this, several risk factors have been identified for persistent OSA post-tonsillectomy.

Recognized risk factors for persistent OSA post-tonsillectomy include children with asthma, allergic rhinitis, age > 7 years, black ethnicity, obesity, syndromic features, or severe pre-operative OSA [14,18,20,28,59]. The severity of the pre-operative OSA is a clinical predictor for residual OSA, with severe OSA defined as an AHI > 4.7 [18,28]. Obesity can also increase the risk of residual post-operative OSA by 3.7-fold [20].

## 8. Beyond Tonsillectomy

Recognizing there is a persistence of OSA in some children after tonsillectomy (with or without adenoidectomy), there is a need to determine what other options of treatment exist for this population. The non-surgical treatment options of medical management; including weight loss, non-invasive home ventilation, and various dental and maxillofacial treatments, are beyond the scope of this article. Adjunct surgical treatments include lingual tonsillectomy, tongue base reduction, uvulopalatopharyngoplasty (UPPP), targeted nasal surgery, supraglottoplasty, epiglottopexy, and tracheotomy [60,61]. Before considering further surgical management, the anatomic location of obstruction needs to be determined. To better understand the level of obstruction in the pediatric airway post-tonsillectomy, the airway can be assessed in sleep. This has been undertaken in several ways; flexible awake laryngoscopy, drug-induced sleep endoscopy (DISE), and sleep cine-magnetic resonance imaging (MRI).

### 8.1. Diagnosis of Level of Obstruction

In patients with persistent OSA following tonsillectomy, there are a variety of ways to assess the airway to determine the level of obstruction. The most straightforward way to assess this is awake flexible laryngoscopy, though this is done in an awake patient, whereas OSA is a disorder of sleep. This is helpful in assessing static structures such as the nasal turbinates, septal deviation or residual adenoids. When trying to assess the dynamic aspects of airway obstruction in sleep, however, this is often insufficient. Assessing the airway in sleep is challenging as this involves either instrumentation of the airway, or real time (cinematic, also known as cine) MRI, neither of which can be easily completed during natural sleep in children [62]. Inducing sleep with anaesthetic agents is then needed.

The optimal sedation for DISE and cine-MRI maintains spontaneous ventilation on room air, and mimics normal sleep in a repeatable manner. It should reproduce the various stages of sleep including rapid eye movement, preserve brainstem reflexes, maintain respiratory rhythm and upper airway muscle activity, yet be relatively short with amnestic properties [13]. Most experts agree that either propofol or dexmedetomidine are preferrable in achieving this [59], although debate continues for the role of other agents [13,63,64].

### 8.2. Drug-Induced Sleep Endoscopy

Drug-induced sleep endoscopy is the flexible endoscopic evaluation of the airway from the nose to hypopharynx in a sleeping patient. This procedure was initially described in 1991 and has been widely adopted in adults to assess the soft palate, oropharynx, tongue base, and epiglottis in sleep [59]. This technique has increasingly been applied to pediatric OSA in the last decade; however, the evidence for its use is still evolving. Controversy still exists in the appropriate indications, sedation regimen, endoscopy protocol, and interpretation of DISE results [13,59,63]. There is strong consensus that a PSG confirming OSA should be completed prior to considering DISE [59,64]. It is generally recommended that DISE is used when there is persistent PSG-proven OSA post adenotonsillectomy [29,59,63,64] as DISE was not found to change the plan for adenotonsillectomy in >95% of surgically naïve children with OSA [59].

The protocol for DISE airway assessment once the child is asleep has largely been standardized [64]. Nasal decongestants are avoided to minimize alterations in nasal airflow. The airway is then assessed endoscopically from nose to hypopharynx [29,61]. Examination of the trachea and bronchi is not routinely recommended as <5% of children studied had lower airway obstruction. Of these, only 0.3% required additional intervention for the lower airway findings [59]. Debate continues, however, on the best method of scoring DISE findings with a goal of predicting surgical outcomes [59,63,64]. Potential complications of DISE include laryngospasm, bronchospasm, oversedation, hypotension, bradycardia, and respiratory depression. As such, this is typically conducted in the operating room with access to skilled personnel, emergency airway equipment, and cardiopulmonary monitoring [59].

### 8.3. Cine-Magnetic Resonance Imaging

In cine-MRI, following sedation with the child breathing spontaneously, a fast echo gradient sequence MRI with axial and midline sagittal sequences is completed in real-time. This method provides high resolution, and dynamic airway evaluation of multiple anatomic sites simultaneously [59,61]. Movement >5 mm at the level of the nasopharynx, posterior oropharynx, or hypopharynx in children with PSG-documented OSA is considered diagnostic of obstruction [62]. Despite being first reported for assessing the airway in sleeping children in the early 2000s [62], there has not been wide adaption of this method across pediatric centers internationally [59].

### 8.4. Beyond Tonsillectomy—Other Surgical Techniques

#### 8.4.1. Lingual tonsillectomy

Lingual tonsillar hypertrophy and hypopharyngeal obstruction is the most frequently noted cause of persistent airway obstruction in children post tonsillectomy [60]. In children with lingual tonsil hypertrophy, removal of most of the hypertrophied tissue can alleviate the point of obstruction [61]. In 2017, a systematic review and meta-analysis of children with persistent OSA had a reduction in the AHI and improvements in their oxygen saturations following lingual tonsillectomy [60]. Surgical methods typically include electrocautery or coblation. Risks of lingual tonsillectomy are similar to those of palatine tonsil surgery, with bleeding and airway complications being most concerning [60]. Lingual tonsillectomy is a viable option in selected patients.

#### 8.4.2. Targeted Nasal Surgery

In patients with persistent OSA, particularly for patients with nasal obstruction, nasal allergies, or those who cannot tolerate CPAP, nasal surgery may be beneficial. This typically includes revision adenoidectomy, inferior turbinate reduction, septoplasty, or some combination of these procedures. Sinus surgery is rarely required. The aim of surgery is to reduce obstruction to facilitate both medical management of any underlying allergies, and improve nasal airflow [61].

#### 8.4.3. Uvulopalatopharyngoplasty (UPPP)

Uvulopalatopharyngoplasty was first described in 1991 [65]. This procedure is typically reserved for children with neurologic impairment and moderate to severe OSA on PSG [61]. This is the extensive restructuring of the soft palate and pharyngeal walls. In this procedure, the palatine tonsils are removed entirely (TT) if still present, with portions of the anterior pillar musculature (palatoglossus) removed. The uvula is resected, and a portion of the soft palate mucosa and muscle (levator palatini) are resected. The soft palate is reapproximated and the tonsillar pillars sutured closed. This results in the posterior tonsil pillar (palatopharyngeus muscle) being pulled anteriorly and superiorly [65]. Post-operative complications include edema, airway obstruction, velopharyngeal insufficiency, and nasopharyngeal stenosis [65]. There is limited data available on the outcomes of UPPP in this patient population.

#### 8.4.4. Supraglottoplasty and Epiglottopexy

Collapse of the supraglottis on inspiration is seen in laryngomalacia. Congenital laryngomalacia is the most common cause of neonatal stridor and may present with failure to thrive and respiratory distress with feeding or sleep [29,61]. Sleep-exclusive laryngomalacia is less common (3.9% incidence), is often occult, and typically presents in older children [29]. The anatomic area of obstruction can be addressed with surgery. This may be division of the aryepiglottic folds (aryepiglottoplasty), removing redundant arytenoid mucosa (arytenoplasty), removing redundant mucosa of the epiglottis (epiglottoplasty), or some combination of these procedures.

Occasionally retroflexion of the epiglottis, either alone or in conjunction with laryngomalacia, may be noted on endoscopic evaluation or DISE. In these patients, the epiglottis needs to be fixated to the base of tongue with epiglottopexy [29]. This is done by denuding the mucosa of the base of the tongue with a partial lingual tonsillectomy and denuding the lingual surface of the epiglottis to promote scarring and to move the epiglottis anteriorly. It may be secured with sutures to fix the epiglottis in place. Post-operative results are confounded with the simultaneous completion of lingual tonsillectomy. Zalzal et al. showed lower AHI post-operatively in patients with PSG-proven OSA and supraglottic collapse following epiglottopexy [29].

#### 8.4.5. Other Surgical Options

Tongue base reduction and tongue base suspension are rare procedures in children. Most children with OSA requiring this intervention have underlying congenital syndromes including severe micrognathia. This is generally reserved for children with persistent OSA after adenotonsillectomy and other failed medical options. In addition to the peri-operative risks of bleeding and airway obstruction, neurovascular damage of the tongue is also a risk [61].

Hypoglossal nerve stimulation is done with an implantable device that stimulates the tongue in time to respiratory effort. This results in protrusion of the tongue with inspiration. This uncommon procedure is effective in selected pediatric populations, typically in children with Trisomy 21 with macroglossia with persistent OSA post-adenotonsillectomy who cannot tolerate CPAP [61].

Finally, tracheotomy is reserved for recalcitrant severe pediatric OSA. Occasionally it may be the first line treatment in children with severe micrognathia, macroglossia, other craniofacial disorders, or severe morbid obesity [61]. This procedure bypasses the entire upper airway, resolving all anatomic levels of obstruction above the tracheotomy tube.

## 9. Conclusions

Tonsillectomy is an ancient procedure that, with the advent of anaesthesia and newer methods, is in wide use worldwide today. To date, no single method has proven to be clearly better overall for cost, risks, complications, or outcomes. It is a very effective procedure to treat childhood OSA; however, when OSA persists post-tonsillectomy, other surgical options may be feasible.

## Data Availability

Not applicable.
